# Integrated Immunization Information System in Indonesia: Prototype Design Using Quantitative and Qualitative Data

**DOI:** 10.2196/53132

**Published:** 2023-12-14

**Authors:** Muhamad Adhytia Wana Putra Rahmadhan, Putu Wuri Handayani

**Affiliations:** 1 Faculty of Computer Science University of Indonesia Depok Indonesia

**Keywords:** vaccination, immunization, immunization information system, high-fidelity prototype, Indonesia

## Abstract

**Background:**

As the volume of immunization records increases, problems with fragmented records arise, especially since the majority of records in developing countries, including Indonesia, remain paper based. Implementing an immunization information system (IIS) offers a solution to this problem.

**Objective:**

In this study, we designed an integrated IIS prototype in Indonesia using the design science research (DSR) methodology.

**Methods:**

The stages of the DSR methodology followed in this study included identifying problems and motivating and defining objectives for a solution, design and development, demonstration, evaluation, communication, and drawing conclusions and suggestions. Specifically, this study began with problem formulation and a literature review. We then applied quantitative (questionnaire with 305 members of the public) and qualitative (interviews with 15 health workers including nurses, midwives, and doctors) data collection approaches.

**Results:**

The resulting high-fidelity prototype follows the 8 golden rules. There are 2 IIS designs, one for the public as immunization recipients and another for health workers. The functionalities include immunization history, schedule, recommendations, verification, certificates, reminders and recalls, coverage, monitoring, news, and reports of adverse events. Evaluation of the prototype was carried out through interviews and a questionnaire designed according to the System Usability Scale (SUS) and Post-Study System Usability Questionnaire (PSSUQ). The SUS value was 72.5 or “Good (Acceptable),” while the system usefulness, information quality, interface quality, and overall value on the PSSUQ were 2.65, 2.94, 2.48, and 2.71, respectively, which indicate it has an effective design.

**Conclusions:**

This provides a guide for health facilities, health regulators, and health application developers on how to implement an integrated IIS in Indonesia.

## Introduction

Infectious diseases such as tuberculosis and malaria are among the top 10 causes of death worldwide [1]. In addition to efforts to treat infectious diseases and relieve symptoms, immunization is another important measure that can prevent infection and thus limit spread among the population [2]. The term “immunization” is often used interchangeably with vaccination or inoculation [3]. Immunization works by training the human immune system to recognize and fight viruses that enter the body by producing antibodies [4]. Recipients are advised to receive all recommended immunization doses in full to receive maximum protection and benefits [5]. According to the World Health Organization (WHO) [6], when health workers administer immunizations, they should (1) determine whether the person meets the requirements to obtain the vaccine, (2) inform the person of the type of vaccine to be given, (3) inform the person of the benefits and side effects that can occur, and (4) inject the vaccine and provide information regarding further doses. If the recipient does not follow a predetermined schedule to obtain a further dose, they are considered to have dropped out of immunization [5].

In Indonesia, data from 2016 revealed that 21 of 34 provinces, such as East Java and DKI Jakarta, have succeeded in minimizing immunization dropout rates in children [7]. However, other provinces, such as Bangka Belitung, South Kalimantan, and Riau, still struggle with such dropouts [7]. Those provinces have child vaccination dropout rates above 5, which is not in accordance with the <5% target set by the Ministry of Health [7]. Therefore, intervention is urgently needed and requires active work from the government, health workers, and the community to continue to reduce immunization dropout rates in children.

Several previous studies have found that health interventions in the form of reminder systems can effectively and significantly help reduce dropout rates from immunization [5,8]. Telemedicine or mobile health applications can also be used to record immunizations. A specific application that stores immunization data and provides reminders for immunization schedules—as well as determines a patient’s immunization status, evaluates public health responses to immunization-preventable diseases, provides information regarding previous immunizations, and facilitates immunization management and accountability [9]—is an immunization information system (IIS). Features often found in an IIS include reminders, records and statuses of previous immunizations, information about immunizations, and appointment management [10].

IIS implementation can be divided into the individual and population levels [11]. At the individual level, information about a person’s immunizations must be recorded consistently and be available throughout the person’s life [11]. Therefore, easy traceability of immunization data is a key requirement. At the population level, the main requirement for an IIS is to have data on the immunization status in certain time periods and geographical areas at the population level [11].

Several previous IIS studies focused on the individual level, with health workers as the users [12-17], while others examined IIS implementation challenges for health workers [13,16,18,19]. However, according to the European Centre for Disease Prevention and Control [11], research on IIS rollout at the individual level should not only focus on health workers but also include the public. Challenges that may arise in the implementation of an IIS or another immunization application need to be identified in advance so that the architecture and prototypes under design can be adjusted accordingly and thus be used sustainably.

Against that background, in the Indonesian context, this study developed an integrated high-level IIS prototype covering all immunization types from childhood to adulthood. To do so, we applied the design science research (DSR) approach, as defined by Peffers et al [20], which is highly appropriate for research in the field of eHealth, to support the creation of innovative solutions to real-world problems [21]. Furthermore, with regard to information systems, DSR can resolve the following 2 main issues: (1) the role of IT artifacts (ie, byproducts of software development that help describe software’s architecture, design, and function [22]) and (2) the implementation of stages in the software development lifecycle. Design activities are at the core of scientific disciplines such as health informatics, and designers should endeavor to develop mobile health that is not only useful but also safe and wholly effective for users [23]. Accordingly, the results of this study can be used to guide health application developers’ work and thus contribute practically to developing an integrated IIS architecture and high-fidelity prototype.

## Methods

### Study Design

First, a literature search was carried out using online databases such as Scopus, Science Direct, IEEE Xplore, and PubMed. The terms or keywords used to search for literature were (“immunization information system” OR “vaccination information system” OR “immunization registry” OR “vaccination registry”). The inclusion criteria were full-text, English-language research articles with a focus on IIS implementation, common IIS functionalities, and potentially discussing implementation challenges.

This study used 2 data collection methods, namely qualitative interviews and a quantitative questionnaire. Respondents were members of the public with knowledge of the health sector, especially regarding immunization, and health workers willing to be involved (purposive sampling). The health workers consisted of nurses, midwives, and doctors who were working in the hospital or clinic, and members of the public included pregnant women and mothers with babies.

We applied DSR, which was implemented in 3 iterations. The use of DSR aims to solve existing problems in society using scientific theory and methods to create solutions that can be applied practically and contribute to theory or science [20]. The DSR stages include identifying problems and defining objectives for a solution, design and development, demonstration, evaluation, and communication. Prototype design was carried out by referring to the 8 golden rules: strive for consistency, offer informative feedback, enable shortcuts for frequent users, design dialogs to yield closure, permit easy reversal of actions, offer error prevention and simple error handling, support internal locus of control, and reduce short-term memory load [24]. The first iteration produced an artifact in the form of an integrated mobile IIS design for Indonesian users. The second iteration produced an artifact in the form of a low-fidelity prototype design. Finally, the third iteration produced an artifact in the form of a final high-fidelity prototype.

A designed artifact should be demonstrated to users before its next iteration is developed. At this stage, demonstrations may be carried out by involving users in experiments, simulations, case studies, evidence review, or other appropriate activities. In this study, for this stage, the questionnaire was prepared and validated. This was prepared with reference to the evaluation approach explained in the following section, and it was then validated through a readability test with the respondents, which ensured each point of a questionnaire statement was correct and easily understood.

**Figure 1:**
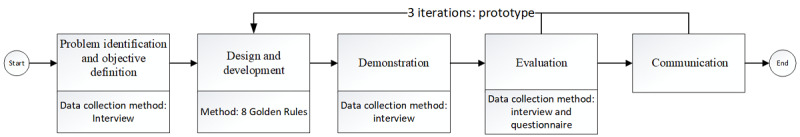
Research steps.

First, we demonstrated the low-fidelity prototype produced in the second iteration. Its evaluation was carried out through semistructured interviews with respondents from the public and health workers. Based on their answers, we developed the high-fidelity prototype as the third iteration. That was then demonstrated, and a quantitative evaluation approach was applied in which responses were gathered through our developed questionnaire. Figure 1 outlines the research steps.

### Research Instruments

We applied an instrument in the form of a questionnaire in the evaluation stage of the third iteration. The questionnaire was structured in several parts. The first contained demographic questions and some questions on the respondent’s immunization experience and their use of immunization applications. The next part contained questions related to the research, namely general prototype improvement questions and others following the System Usability Scale (SUS) and Post-Study System Usability Questionnaire (PSSUQ) approaches to evaluate the application design. In the SUS approach (Multimedia Appendix 1), questions were answered using a 5-level Likert scale (1=strongly disagree, 2=disagree, 3=neutral, 4=agree, 5=strongly agree), while the PSSUQ (Multimedia Appendix 1) used a 7-level Likert scale (1=strongly agree, 2=agree, 3=slightly agree, 4=neutral, 5=strongly disagree, 6=disagree, 7=strongly disagree). The qualitative interview guide is also included in Multimedia Appendix 1.

### Data Analysis

Qualitative data derived from interviews were analyzed using content analysis. With this method, interview responses are categorized, and each category is reviewed and either accepted or rejected for implementation in the next iteration of application design. That decision is based on the number of similar responses (ie, carrying forward those with the highest numbers of votes [majority]). Meanwhile, data derived from the results of the questionnaire (with SUS and PSSUQ parts) were analyzed using mathematical calculation methods. The final SUS scores (in the range of 0 to 100) were obtained using the formula developed for the approach by Bangor et al [25]. This “Adjective Ratings” measurement rule represents the resulting SUS scores in specific categories using qualitative statements (non-numbers). The scores were then compared with the predetermined measurement levels to determine the usability level of the application design. The PSSUQ is an instrument designed to assess user satisfaction with a computer system or application and consists of 16 statement items [26]. These produce 4 scores, namely 1 overall score and 3 subscale factors: system usefulness, information quality, and interface quality [27]. The overall score is the average of all items, system usefulness represents the average of items 1 to 6, information quality represents the average of items 7 to 12, and interface quality represents the average of items 13 to 15 [28]. Lower PSSUQ scores indicate higher levels of satisfaction; final scores are obtained by calculating the average Likert scale score for each statement.

### Ethical Considerations

All respondents agreed to participate in this study, which received approval from the Faculty of Computer Science, University of Indonesia (reference number: S-254/UN2.F11.D1.5/PPM.00.00/2023). All respondent data were anonymous and could only be used for the purposes of this research.

## Results

### Identify Problem and Define Objectives of a Solution

Problem identification and determining the objectives of the solutions to be built required seeking information regarding the conditions of immunization implementation in Indonesia. In the first stage, an information search was carried out using various trusted news channels such as the national news provider. In addition, a search was also carried out through various literature sources, in the form of journal articles, to gain insights from previous research. In the next stage, interviews were conducted with health workers and the public to identify problems in the field. Interviews were held online with 15 health workers as well as members of the public including mothers with babies. From 15 interviews, we reached saturation at which enough data had been collected to draw conclusions [29]. The interviews were conducted from February 11, 2023, to February 22, 2023, via online meeting platforms. Demographic details of the health worker respondents can be seen in Table 1, while those for the public respondents can be seen in Table 2. All respondents in Table 2 were undergraduates in a bachelor program.

Interviews were conducted to obtain in-depth information regarding the implementation of immunization in Indonesia, immunization challenges faced by health workers and the general public, respondents’ experiences with using immunization applications, respondents’ opinions regarding applications’ required functionalities, data that must be stored in these applications, and respondents’ expectations of them. Table 3 summarizes the results of those interviews, with responses categorized according to our content analysis.

Summaries of the questionnaire results on application functionality from the perspectives of the general public and health workers can be seen in Table 4 and Table 5, respectively. Table 6 summarizes the data that must be stored in the application.

**Table 1:** Demographics of health worker respondents.

Respondent	Gender	Age (years)	Education	Role	Organization	Years of service
TK1	Female	52	Midwifery bachelor	Midwife	Community health centers	30
TK2	Female	31	Midwifery diploma	Midwife	Hospital	10
TK3	Male	31	Midwifery diploma	Nurse	Community health centers	4
TK4	Male	30	Midwifery bachelor	Nurse	Community health centers	4
TK5	Female	30	Midwifery diploma	Midwife	Hospital	3
TK6	Female	23	Medical profession	Doctor	Community health centers	1

**Table 2:** Demographics of public respondents.

Respondent	Gender	Age (years)	Occupation
MU1	Female	36	Civil servant
MU2	Female	30	Private employee
MU3	Female	35	Civil servant
MU4	Female	24	Private employee
MU5	Male	22	Private employee
MU6	Female	24	Private employee
MU7	Male	24	Private employee
MU8	Female	22	Private employee
MU9	Male	22	Private employee

**Table 3:** Results of content analysis interviews conducted for problem identification.

Category and Problem (respondent ID)		Feature recommendation
**Immunization information**		
	Immunization information difficult to access, especially concerning immunization for adults (MU1, MU2, MU4, MU5, MU6, MU7, MU9, TK5)	Immunization news articles and features regarding immunization side effects (AEFI^a^)
	The existence of antivaccine groups due to lack of information about the benefits and side effects of immunization (MU1, MU2, MU5, TK1, TK4)	Immunization news articles and features regarding immunization side effects (AEFI)
**Implementation of immunization**		
	Forget immunization history (MU1, MU2, MU3, MU4, MU5, MU6, MU7, MU8, MU9, TK1, TK2, TK5, TK6)	Certificate features and digital immunization history records
	Missing, lost, or damaged records (certificates) of immunization history (MU1, MU2, MU4, MU5, MU6, MU7, MU8, TK1, TK2, TK6)	Certificate features and digital immunization history records
	Information on side effects of immunization and how to deal with them (MU1, MU2, MU4, MU9)	Features regarding immunization side effects
	The need for a centralized and integrated immunization application (MU1, MU5, MU6, MU7, MU9, TK3, TK4)	Integrated application design
	The need for a reminder mechanism for immunization (MU1, MU2, MU3, MU4, MU5, MU6, MU8, TK2, TK4, TK5, TK6)	Reminder and recall feature

^a^AEFI: adverse events following immunization.

**Table 4:** Scores on the questionnaire about application functionality with the public as users.

Application functionality	Score, mean
Access to immunization information	4.78
Access to immunization history	4.67
Reporting of adverse postimmunization events or side effects after immunization	4.67
Immunization certificate	4.44
Display of immunization information that has not been received	4.33
Reminder or recall notifications	4.22
Immunization recommendations	4.11

**Table 5:** Scores on the questionnaire about application functionality with health workers as users.

Application functionality	Score, mean
Record of patient immunization information	4.50
Monitoring of patient adherence to the immunization schedule	4.50
AEFI^a^ reporting and vaccine safety monitoring	4.50
Production or verification of immunization certificates	4.33
Immunization coverage information	4.33
Immunization data exchange with other health information systems	4.17

^a^AEFI: adverse events following immunization.

**Table 6:** Required immunization information system (IIS) data.

Data		Public, mean	Health workers, mean
**Immunization recipient data (children or adults)**			
	Citizen ID number	4.50	4.83
	Name	5.00	4.67
	Gender	5.00	4.67
	Date of birth	5.00	4.67
	Place of birth	4.75	4.00
	Home address	4.25	4.67
	Weight and height	4.75	4.67
	Allergy	4.25	4.33
	Photo	3.50	3.17
	Phone number	4.75	4.33
	Email	4.75	3.00
**Accompanying data (eg, data on parents of children receiving immunizations)**			
	ID number	4.25	4.33
	Name	4.75	4.33
	Status	4.75	4.67
	Gender	4.50	4.33
	Date of birth	4.00	3.67
	Email	4.25	3.00
	Phone number	4.25	4.17
	Address	4.25	4.17
**Immunization data**			
	Health facility name	4.75	4.67
	Health facility address	4.75	4.33
	Health facility phone number	4.25	4.67
	Health facility email	3.75	3.83
	Type of vaccine	5.00	4.83
	Immunization date	4.75	4.83
	Vaccine batch number	0	4.83

### Design and Development

At the individual level, IIS functionality can be grouped into 2 modules, namely clinical decision support and report generation [11], while at the population level, IIS functionality can be grouped into 3 modules, namely immunization uptake monitoring, communication, and safety monitoring (Table 7). In the clinical decision support module, functionality consists of immunization history, schedule, recommendations, and reminders and recalls. In the report generation module, the functionality consists of immunization certificates. In the uptake monitoring module, the functionality consists of immunization coverage and monitoring. In the communication module, the functionality consists of immunization news. In the safety monitoring module, the functionality consists of adverse events following immunization (AEFI).

**Figure 2:**
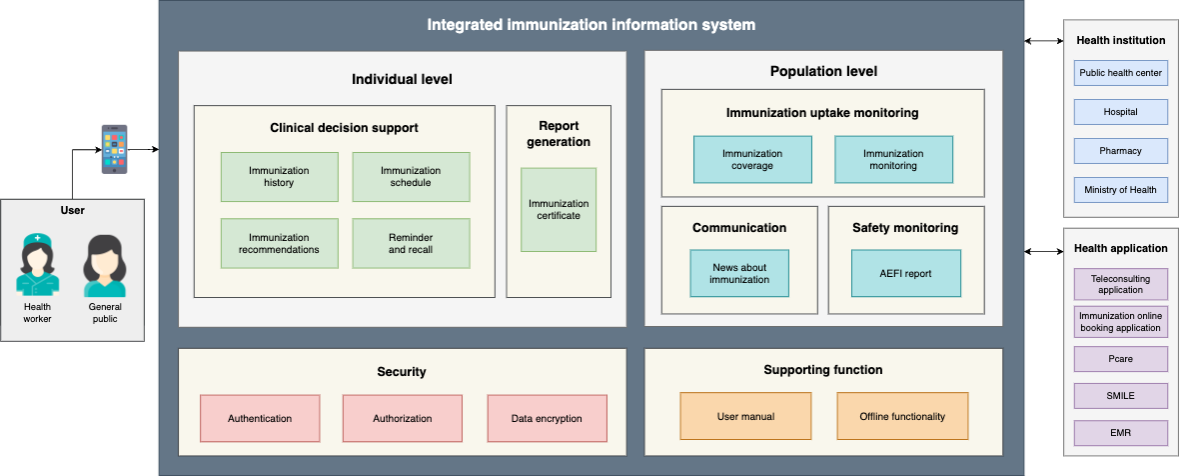
Integrated immunization information system (IIS) model in Indonesia. AEFI: adverse events following immunization; EMR: electronic medical record.

Figure 2 summarizes the proposed IIS system model, which shows integration with health information systems and health service providers in Indonesia. Specifically, several parties, such as health facilities, pharmacies, and the Ministry of Health, must be integrated with the IIS. Meanwhile, health facilities are integrated through electronic medical records (EMRs) in the hospital information system or health community information system, which facilitates clinical decision support and report generation as health facilities can see a patient’s immunization history, as well as supporting immunization recommendations based on the patient’s allergies recorded in their EMR.

**Table 7:** Description of immunization information system (IIS) functionality.

Level, module, and functionality					Description
**Individual level**					
	**Clinical decision support**				
		Immunization history		Record and display immunization history information	
		Immunization schedule		View immunization schedules	
		Reminder and recall		Immunization schedule reminder notifications	
		Immunization recommendations		Immunization recommendations offered	
	**Report generation**				
		Immunization certificate		Verify and view immunization certificates	
**Population level**					
	**Immunization uptake monitoring**				
		Immunization coverage		Monitor acceptance of immunization in the community	
		Immunization monitoring		Monitoring community compliance with the immunization schedule	
	**Communication**				
		News on immunization		Dissemination of up-to-date information about immunization	
	**Safety monitoring**				
		AEFI^a^ report		View AEFI reports	
	**Security**				
		Authentication		User authentication to gain access on IIS	
		Authorization		Allow access to IIS information to users	
		Data encryption		Secure sensitive information stored on IIS	
	**Supporting function**				
		User manual		Describe the features of IIS	
		Offline functionality		Data synchronization when online and offline	

^a^AEFI: adverse events following immunization.

Furthermore, the IIS is integrated with pharmacies via the SMILE application, offering information on a patient’s vaccines, such as the vaccine type, batch number, and side effects. This integration is needed in the safety monitoring module. Additionally, the IIS is integrated with other mobile health applications such as Pcare and SatuSehat to exchange further immunization data, especially regarding a patient’s COVID-19 immunization history, and to provide teleconsultation features and online immunization booking. Furthermore, the IIS is integrated with the national health data repository, which is used by the Ministry of Health to analyze health data in Indonesia, through the immunization uptake monitoring and communication module.

The IIS application developed will be accessed by users via the internet; therefore, a firewall secures the connection. Furthermore, the application programming interface (API) is used as the medium for interaction between the IIS and other information systems. The API is a data exchange mechanism commonly used by health service providers in Indonesia and is an interoperability solution recommended by the Ministry of Health through the health digital transformation strategy [30]. Accordingly, it follows technological principles around interoperability.

The API used in this research is Fast Health Interoperability Resources (FHIR), which can be used to manage electronic record systems such as an IIS. It is the latest generation standard framework created by Health Level 7 (HL7) [31]. FHIR is an international data exchange standard recommended by the WHO, for example, for COVID-19 immunization certificates [32]. FHIR is also recommended by the Ministry of Health to resolve data exchange problems in health information systems in Indonesia [30]. FHIR is flexible and can be adapted to the needs of stakeholders.

### Low-Fidelity Prototype Development

For the general public, there are several main features required in an application, such as immunization features (schedules, detailed immunization information, and immunization certificates) that include child, adult, and COVID-19 immunizations; AEFI reporting; immunization news articles; and reminder and recall notifications.

**Figure 3:**
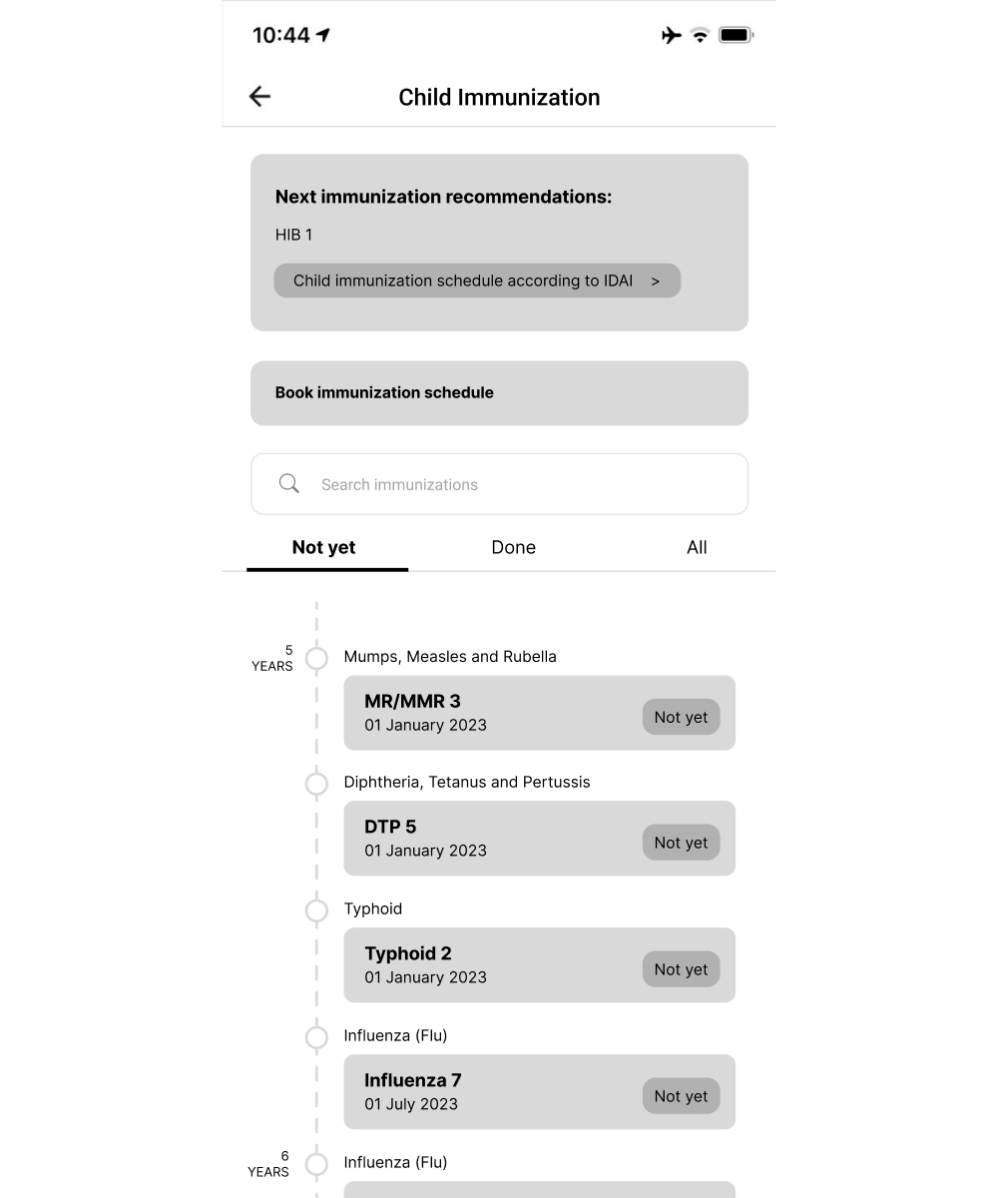
Child immunization page in the second iteration of the immunization information system (IIS) application.

The child feature contains immunization information for those aged up to 18 years, including a list of immunizations children can receive, the recommended timings of those immunizations, and recommendations based on the specific child’s age. For example, when a baby is a newborn, they should immediately be immunized against hepatitis B 1, polio 0, and bacille Calmette-Guérin (BCG; according to the Indonesian Pediatrician Association). The application uses a timeline display, so that parents can easily monitor the progress of their child’s immunizations. In addition, the filters “Not yet,” “Already,” and “All” make it easy for parents to see their child’s immunization status. Users can also search for an immunization through the search feature provided. The display of the child immunization page can be seen in Figure 3.

For health workers, there are several main features required in an application, such as an immunization recording feature for child, adult, and COVID-19 immunizations; AEFI reporting; immunization coverage; and immunization monitoring. Each of these is explained herein. The immunization recording feature assists health workers with recording the public’s immunization histories, covering their child, adult, and COVID-19 immunizations. This feature consists of 2 main pages, namely the immunization recipient list and detail pages.

**Figure 4:**
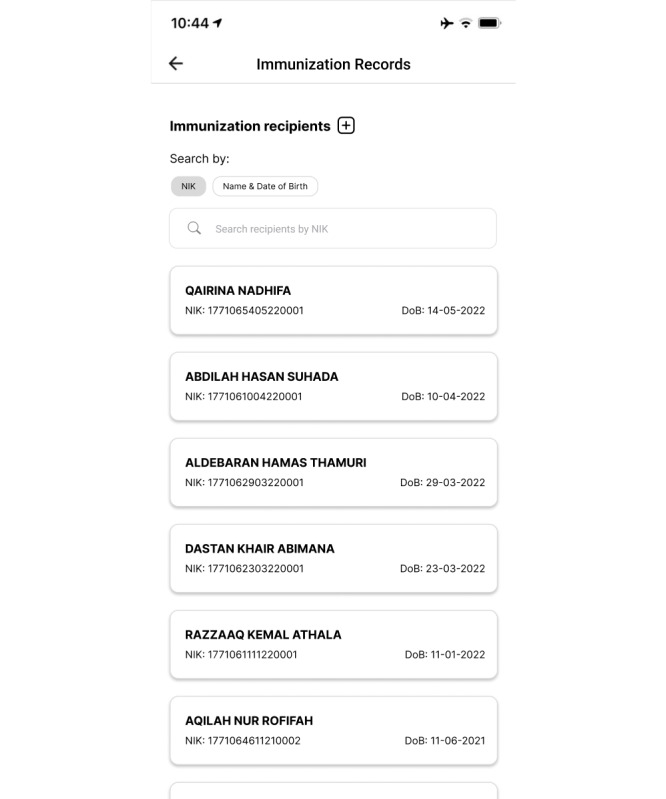
Immunization record list page in the second iteration of the immunization information system (IIS) application.

On the immunization recipient list page, members of the public are listed (name, citizen identification number [NIK], and date of birth) if they are within the scope of the health worker’s facility. Health workers can search among community data using the search bar provided by typing the NIK, name, or date of birth of an immunization recipient. They can add data via the “+” button. The appearance of the list of immunization recipients can be seen in Figure 4.

**Figure 5:**
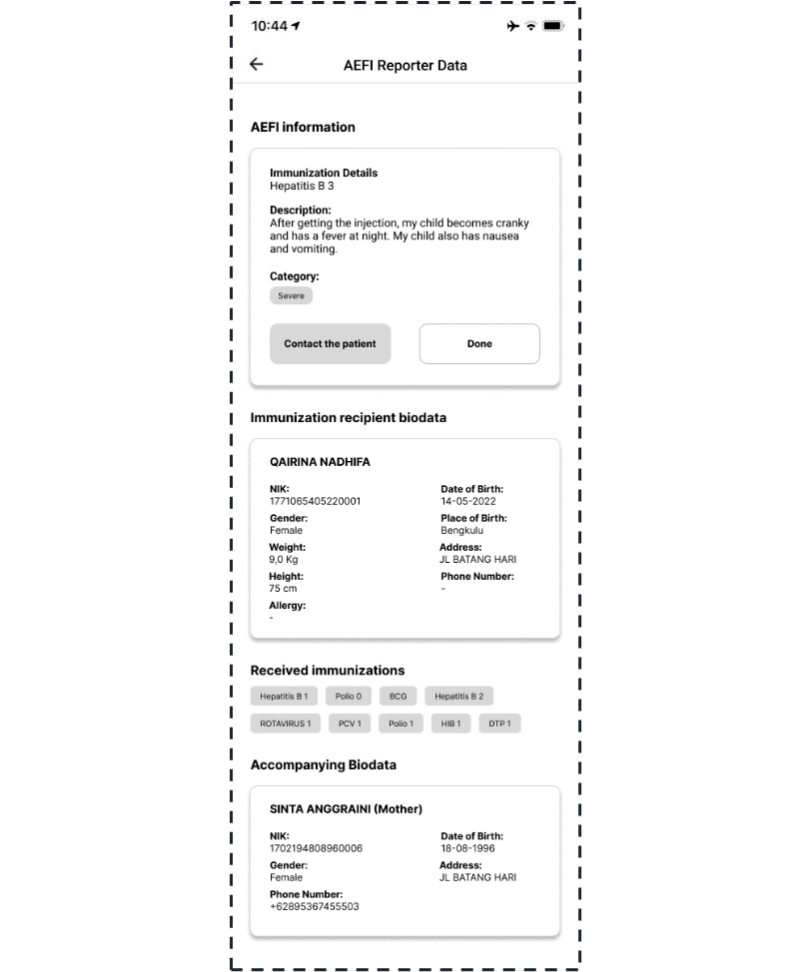
Adverse events following immunization (AEFI) report page in the second iteration of the immunization information system (IIS) application.

Imagine a scenario in which a health worker is following up on members of the general public who have reported side effects following immunization. If the health worker chooses a name from the list provided, they will be directed to the AEFI report page containing the individual’s report (such as the vaccine’s name, the symptoms experienced, and the severity level), along with other information that may be relevant such as biodata and history of immunizations. The health worker can then follow up on the report by contacting the individual via phone, SMS text messaging, or WhatsApp chat to advise how to manage their symptoms. Once followed up, the health worker can press the “Done” button. The display of this page can be seen in Figure 5.

### High-Fidelity Prototype Development

**Figure 6:**
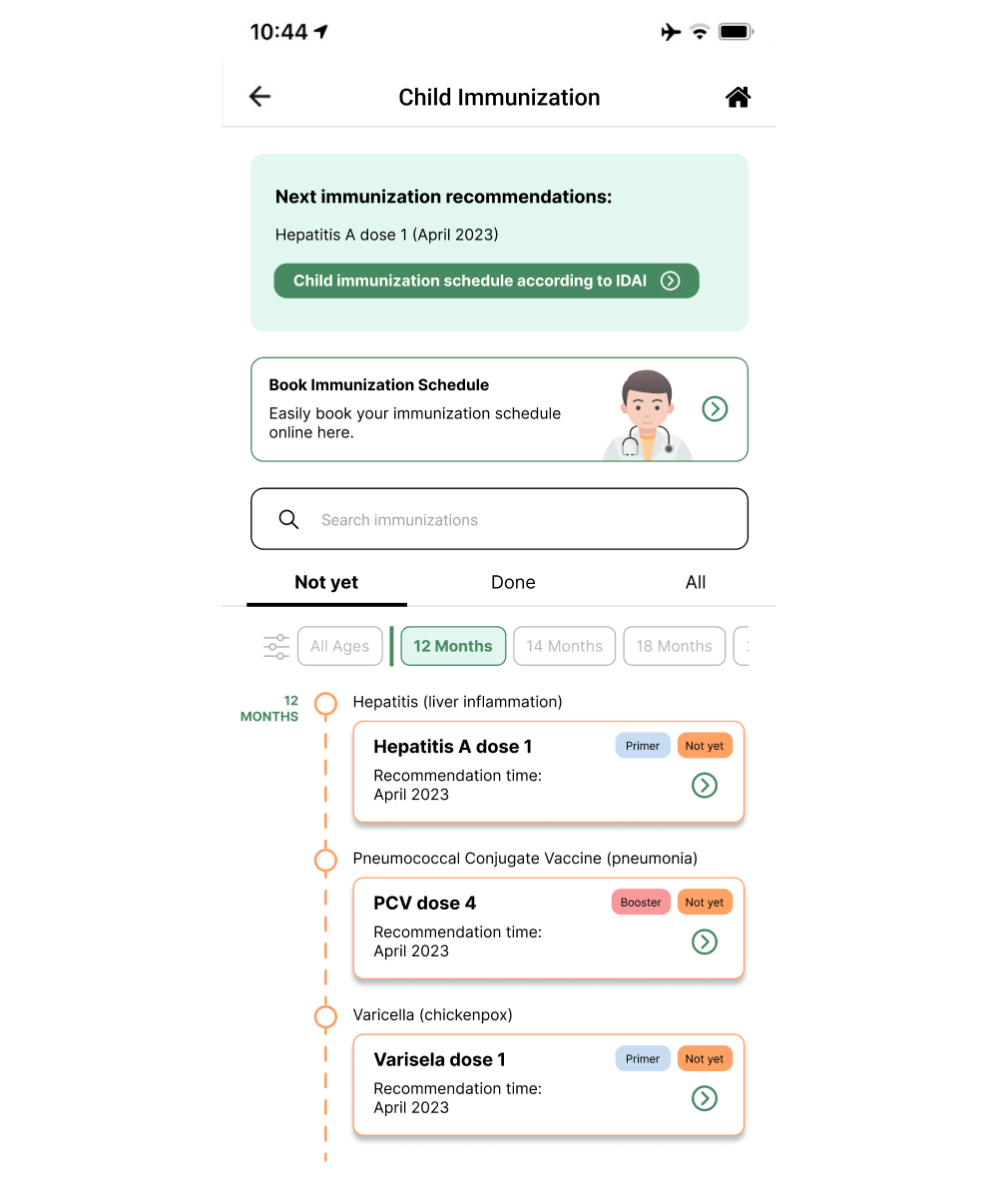
Child immunization page in the third iteration of the immunization information system (IIS) application.

**Figure 7:**
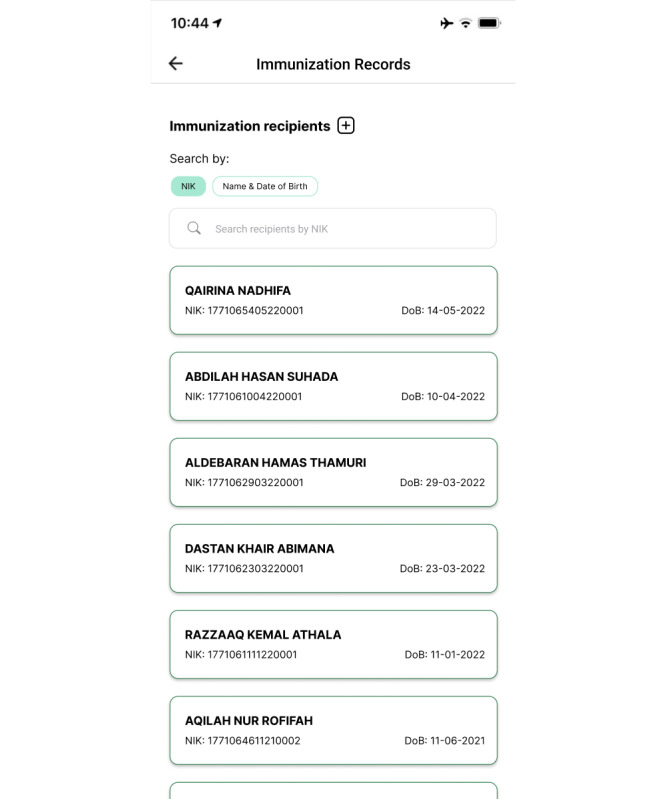
Immunization recording page in the third iteration of the immunization information system (IIS) application.

When improving on the second iteration as we began to prepare the third iteration, the first changes we made were to the child immunization page, which included adding immunization recommendations, changing the button design for booking immunizations, adding an immunization age filter, adding information on primary and booster statuses of immunizations, and adjusting the date display. The new appearance of this page can be seen in Figure 6. In the immunization recording feature, we changed the appearances of the immunization recipient list and detail pages. On the former, the changes made were minor, namely adding color to the display. On the latter, there was a change in the composition of the information displayed. Rather than displaying the individual’s bio in full on this page, in the third iteration, we created a special page to display their bio without repetition between features. The display of this page can be seen in Figure 7.

**Figure 8:**
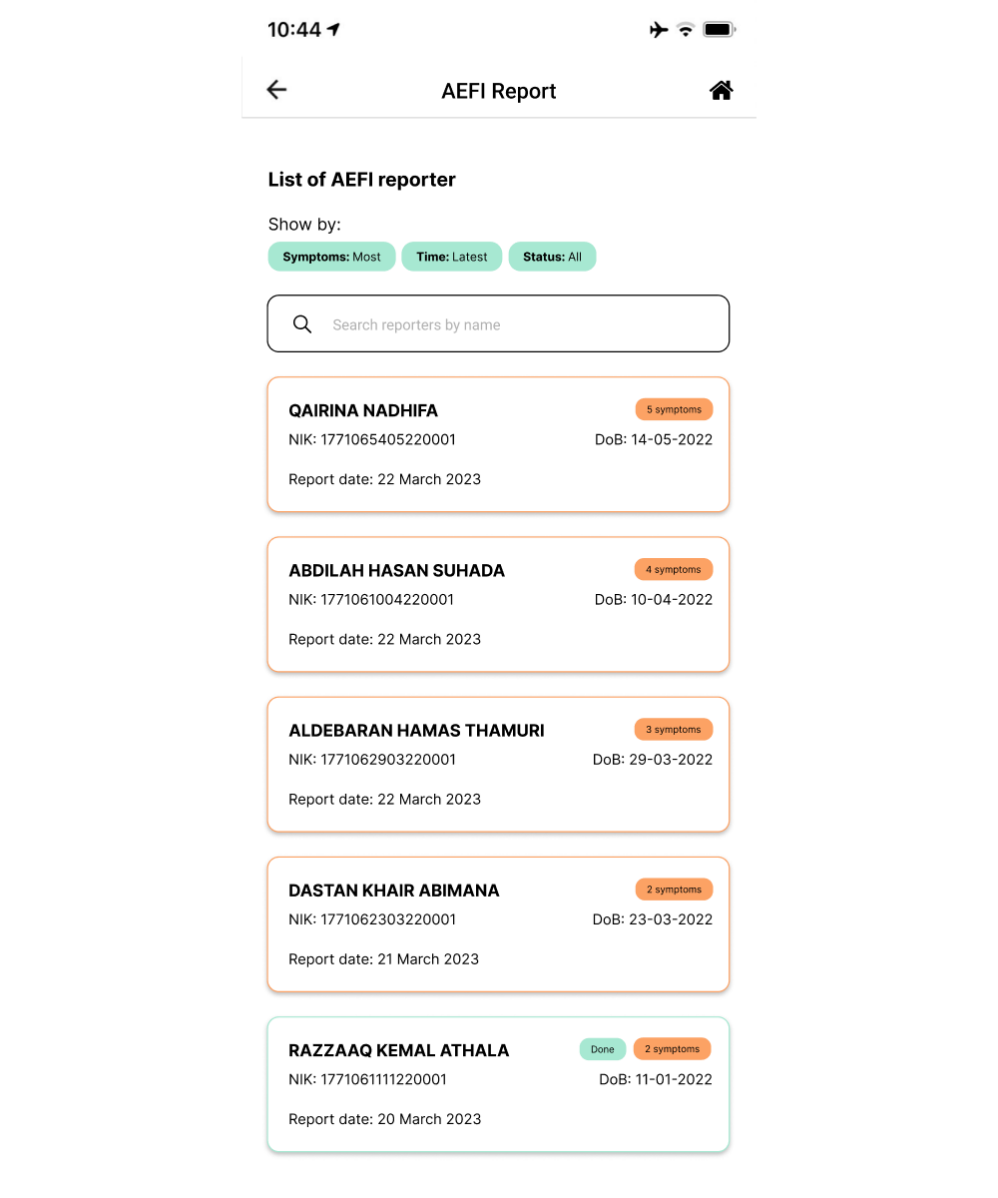
Page list of adverse events following immunization (AEFI) reports in the third iteration of the immunization information system (IIS) application.

Furthermore, we changed the appearances of the AEFI reporting list and detail pages. On the former, we removed the filter for the severity of symptoms reported by members of the general public, to reduce the impact of bias in reports. The new filter options were the number of symptoms, time of report, and status of report. The display of this page can be seen in Figure 8.

### Demonstration, Evaluation, and Communication

In preparing these iterations of the IIS, demonstration and evaluation stages were carried out in 2 ways. The first was by presenting the low-fidelity prototype design to the public and health workers. The presentation was carried out via an interview with each respondent. Interviews were conducted with 11 respondents consisting of 6 members of the public and 5 health workers. The online interviews were conducted from March 29, 2023, to April 9, 2023. Demographic details of the health worker interviewees can be seen in Table 1, while demographics of the general public interviewees are in Table 2.

The second demonstration was of the high-fidelity prototype design, which was assessed through an online questionnaire on the Survey UI platform. This was distributed to the general public in Indonesia without any specific criteria, using the random sampling method, via social media applications such as Line, WhatsApp, and Telegram. The questionnaire was distributed from April 23, 2023, to May 17, 2023. In total, 305 responses were obtained; the demographic details for the respondents can be seen in Multimedia Appendix 1.

The questionnaire evaluation results are summarized in Multimedia Appendix 1. They showed that 294 respondents (294/305, 96.4%) were satisfied with the prototype design. The average SUS score, according to the measurement rules established by Bangor et al [25], was “Good” (72.5) on the adjective ratings scale. Next, the PSSUQ scores in 4 categories were obtained based on the calculations previously outlined, as shown in Table 8.

**Table 8:** Post-Study System Usability Questionnaire (PSSUQ) final evaluation scores.

Evaluation object	System usefulness, mean	Information quality, mean	Interface quality, mean	Overall, mean
High-fidelity prototype	2.65	2.94	2.48	2.71

## Discussion

### Principal Findings

**Figure 9:**
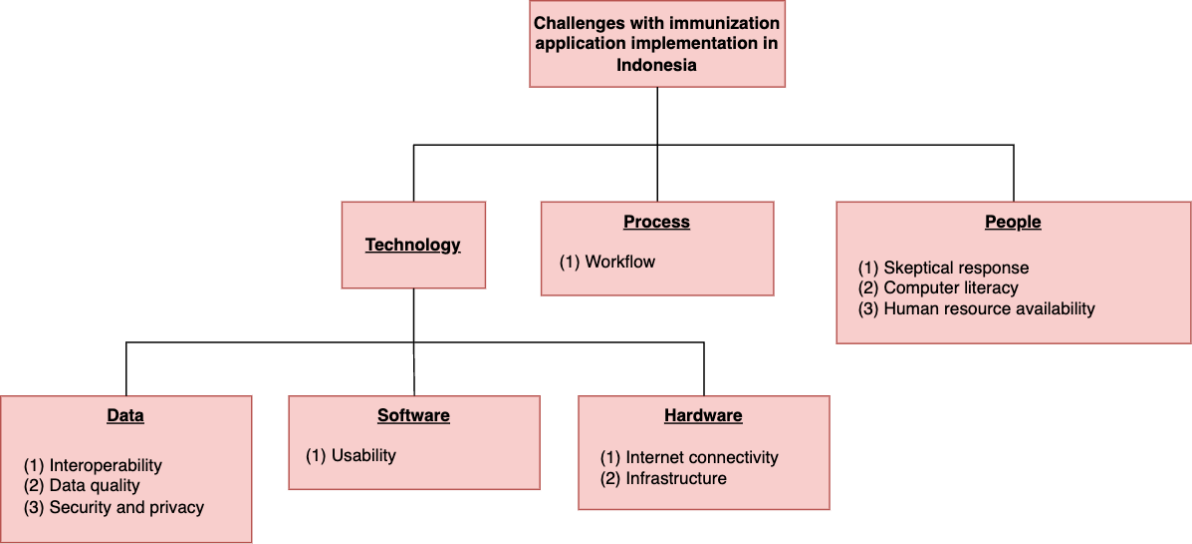
Challenges with implementing an immunization information system (IIS) in Indonesia.

Through our interviews with the general public and health workers, 10 obstacles were identified in the implementation of an IIS, relating to interoperability, quality, security, and privacy, among others. These challenges were grouped into major themes, namely people, process, and technology. The latter was then subdivided into challenges in the fields of software or hardware. An illustration of this breakdown can be seen in Figure 9.

Interoperability between health applications in Indonesia, including immunization applications, is a key challenge to implementing an integrated IIS. Interoperability is the ability of a system to share information with other systems effectively and efficiently [33]. For instance, while some community health centers and hospitals have started using applications, others still use paper-based immunization recording, as described by interviewee TK3:

For children’s immunizations, the recording is still manual using paper...The hope is that child immunization can also be integrated into the same application. For example, if there are parents who immunize their children at the community health centers, but then later immunize them at the hospital through application A, they can be connected to each other.

TK3

As this demonstrates, interoperability is needed for comprehensive and complete community immunization from different health care providers. In addition, such interoperability benefits the government’s epidemiological and surveillance studies, which can be used to establish health policy.

Quality refers to the consistency, accuracy, completeness, and timeliness of the user’s information stored in the IIS [34]. The interviews highlighted that there are children who were not assigned an NIK when they were born, which is especially the case for people who live rurally. New parents may not register their child for an NIK until they start school, when one is required for school registration. This constrains the process of recording immunizations before the school years. Health workers currently overcome this issue by using a parent’s NIK, under which the child’s immunization history is recorded, as described by interviewee TK3:

For children’s immunizations, the recording is still manual using paper. Sometimes you need a family card or a citizen identity number. For example, if the child doesn’t have a citizen identity number, we usually use their parent’s citizen identity number.

TK3

However, this threatens the consistency and accuracy of the information stored in an immunization application.

Health workers also find it challenging to ensure the completeness and timeliness of immunization records since applications often cannot be opened when the internet connection is not smooth or when it drops due to a power outage, as described by interviewee TK4:

If internet access is smooth, if the light is on, it is safe for input. Sometimes, if there is a problem with the electricity or the internet, the input assignments are taken home, where they are recorded via personal cell phone.

TK4

Another consideration is protecting the security and privacy of the users of an IIS [11]. Some interviewees worried and were unsure about the security of their data stored in health applications, following an incident in which data were leaked from the national COVID-19 contact tracing application developed by the RI Ministry of Health in November 2022, as described by interviewee MU8:

I’m worried about the security of my data stored in the health application because last year, our health application was hacked, and billions of users’ data were leaked. The government should pay more attention to the security of stored data.

MU8

Clearly, this harmed public trust in government-developed applications. That mistrust has been exacerbated by citizen information stating sensitive data in the government-developed health apps do not have maximum security protection using data encryption, for example. This is despite the Regulation of the Minister of Communication and Informatics No. 20 of 2016 concerning the Protection of Personal Data in Electronic Systems, which requires that “Personal Data stored in Electronic Systems must be in encrypted data form.”

In the software category, a challenge identified was that of usability, referring to whether information systems can be used effectively and efficiently [35]. The interviews revealed that both health workers and the public experienced obstacles when using immunization applications. For health workers, immunization records could not be edited once saved, such as to correct errors. As interviewee TK4 described:

The application cannot edit data, so once it is done, it cannot be changed anymore. You need to provide a menu for editing, so a maximum of 3 days after the first input, it can still be edited, for example. It should remain editable for a certain time.

TK4

In the hardware category, 2 types of challenges were identified, namely internet connectivity and infrastructure. The former refers to the availability of or access to the internet when using an IIS [36]. The public and health workers alike struggled with this, especially in rural areas where internet access is poor and power outages are frequent. Ideally, an IIS should still operate offline, then send data to the server when the system reconnects to the internet, as described by interviewee MU7:

With internet connectivity constraints, [updates are needed] so the immunization history might be seen offline.

MU7

Infrastructure refers to the availability and capabilities of servers, database structures, and hardware to run an IIS [34]. In our interviews, the main infrastructural constraint mentioned was the availability of an electricity source. Currently, the interviewees use generators as a temporary solution to this challenge as explained by interviewee TK1:

The main obstacle is electricity. WiFi depends on electricity. So, patients sometimes wait half an hour, 1 hour. The solution is temporarily using a generator.

TK1

Aside from a power source, another challenge faced is the availability of hardware such as computers and mobile phones that can access applications. Currently, health workers often still use their personal hardware to record immunizations.

This research has theoretical implications as it fills gaps from previous research regarding the development of an IIS, especially in resource-limited countries. When searching previous research, we found the development and implementation of an IIS and the analysis of challenges experienced, when viewed at the individual level with the public as users, were not discussed extensively [19]. Previous research at the individual level was largely focused on health workers as IIS users [12-17]. However, according to the European Centre for Disease Prevention and Control [11], research on IIS use at the individual level should not only focus on health workers but also include the general public. From the general public’s perspective, this study found that security and privacy, usability, and internet connectivity are the main challenges experienced when using immunization applications.

Furthermore, this research also fills a gap from previous research regarding the interoperability of health applications in resource-limited countries. Previously, studies have been carried out in countries with similar problems and characteristics to Indonesia. This research extended their findings by designing an architectural model for an integrated IIS prototype in Indonesia, the primary focus of which is the needs of the public while still covering the needs of health workers. This prototype incorporated features for the public that were not discussed extensively in previous studies, such as the adult immunization feature, AEFI, and immunization verification; the results obtained from the SUS and PSSUQ analysis represented a good rating. The IIS architectural model and prototype were developed using a DSR approach, which can guide researchers in developing an IIS according to users’ needs.

Thus, this research has practical outcomes in providing guidance for health application developers when working on an IIS for child, adult, and COVID-19 immunizations in resource-limited countries. In particular, it guides health regulators and application developers in considering the functionalities needed, the parties and data sources to be integrated, the technologies that can be used to develop the IIS, and interoperability with other health applications.

Moreover, the prototype design produced in this study is expected to be used as an illustration in future work to develop such an IIS, given it fulfills the 8 golden rules and has good SUS and PSSUQ evaluation scores. Features not yet widely implemented in immunization applications, such as adult immunization, AEFI, and immunization verification, are particularly expected to guide IIS development beyond the primary function to record a child’s immunization history. Another key guiding point is interoperability with other health applications, such as the function of booking immunizations online or sending messages to health workers via linkages to health and teleconsulting applications already used in Indonesia. Furthermore, there are not yet health regulations in Indonesia for the implementation of electronic immunization records via an IIS, but this research contributes to demonstrating their benefits. We hope it will encourage the timely introduction of government regulations for the implementation of electronic immunization records through an IIS, which should prompt their immediate rollout in Indonesia.

This research has several limitations. Respondents to the questionnaire evaluating the IIS prototype made were dominated by respondents aged 19 years to 24 years (154/305, 50.5%), so they did not fully represent potential users of all age ranges. In addition, to further evaluate the integrated IIS model and the prototype developed in this study, future research can carry out IIS implementation with case studies in several health facilities to determine the real-world impact of IIS implementation. Future research can be conducted to analyze further the need for regulations that can become a reference for health facilities or health application developers regarding the implementation of electronic immunization records through IIS.

### Conclusion

In this study, we produced an IIS prototype for the public and health workers. Its features include immunization history, schedule, recommendations, verification, certificates, reminders and recalls, coverage, monitoring, news, and AEFI. Its functionality also spans child, adult, and COVID-19 immunizations. This fills a gap in previous IIS research, which largely focused on child immunization. In addition, the AEFI report feature and immunization verification were not discussed extensively in previous studies. Prototype development was carried out considering the 8 golden rules and evaluating the prototype in 3 iterations. The result obtained from the SUS analysis was a Good (Acceptable) rating, and in the PSSUQ analysis, the IIS prototype achieved a good value overall, as well as specifically for system usefulness, information quality, and interface quality.

## Appendix

Multimedia Appendix 1 Supplementary material.

## Abbreviations

AEFI: adverse events following immunization
API: application programming interface
BCG: bacille Calmette-Guérin
DSR: design science research
EMR: electronic medical record
FHIR: Fast Health Interoperability Resources
HL7: Health Level 7
IIS: immunization information system
NIK: citizen identification number
PSSUQ: Post-Study System Usability Questionnaire
SUS: System Usability Scale
WHO: World Health Organization

## Data Availability

The data sets generated or analyzed during this study are not publicly available due to a lack of authority to share the data.
