# Supplementary material for: Integrated Immunization Information System in Indonesia: Prototype Design Using Quantitative and Qualitative Data
Source: JMIR Form Res. 2023 Dec 14;7:e53132. doi: 10.2196/53132 (PMC10755663; doi:10.2196/53132)
Supplement: Multimedia Appendix 1 [file formative_v7i1e53132_app1.docx]

**SUS Questionnaire Instruments**

| **Code** | **SUS Questions** |
| --- | --- |
| S1 | I think I will use this application often |
| S2 | I think this application is too complex (displays many unnecessary things) |
| S3 | I find this application easy to use |
| S4 | I need technical assistance (tutorial/FAQ) to use this application |
| S5 | I think the features provided in this application are well designed |
| S6 | I judge too many inconsistencies in this application (example: features, images, explanations) |
| S7 | I feel most people will quickly find it easy to use this application |
| S8 | I think this application flow is very complicated to use |
| S9 | I feel very confident using this application |
| S10 | I need to learn many things to use this application properly |

**PSSUQ Questionnaire Instruments**

| **Dimension** | **Description** | **Code** | **PSSUQ Questions** |
| --- | --- | --- | --- |
| System  Usefulness | A system that can be easily learned and used, can effectively complete its tasks, and can quickly increase the productivity of its users | P1 | Overall, I am satisfied with the ease of use of the application. |
|  |  | P2 | I find it easy to use this application. |
|  |  | P3 | I can complete tasks and scenarios fast through this application. |
|  |  | P4 | I feel comfortable using this application. |
|  |  | P5 | I find it easy to learn how to use this application. |
|  |  | P6 | I will be productive using this application. |
| Information Quality | The information provided by the system can be easily understood, can effectively assist in completing tasks and being organized. | P7 | I got an error message explaining how to fix it. |
|  |  | P8 | Whenever I make a mistake in the application, I can easily and quickly fix it. |
|  |  | P9 | I feel the help information provided by the application is very clear. |
|  |  | P10 | I find it easy to find the information needed. |
|  |  | P11 | The information provided by the app was effective in helping me work on assignments and scenarios. |
|  |  | P12 | The information arrangement provided by the application is quite clear. |
| Interface  Quality | The system created can provide satisfaction to its users both in appearance and as a whole. | P13 | I feel the interface is quite comfortable. |
|  |  | P14 | I like the interface in the application. |
|  |  | P15 | I feel that all the functions and capabilities provided by the application have met my expectations. |
|  |  | P16 | Overall, I am satisfied with this application. |

**Interview Instruments**

1. Where do you usually get immunization information?

2. How important is access to immunization information to you?

3. Do you know what immunizations you have received?

4. At what health facility do you usually get immunized?

5. Where is information about your immunization stored?

6. What are your challenges to get immunized?

7. What are your challenges regarding the mechanism for recording immunizations?

8. Do you use health applications such as PeduliLindungi, Halodoc, Alodokter, Primaku, or other applications? What features do you use?

9. What functional requirements do health applications need? Especially with regards to immunization?

10. What information or data would you like to see regarding your immunization history?

11. What challenges do you experience in using the current health applications?

12. What challenges do you experience regarding your child's immunization?

**Demographics of Respondents for Health Workers at the Demonstration Stage in the Third Iteration**

| **Respondent** | **Gender** | **Age** | **Education** | **Occupation** | **Organization** | **Working Period** |
| --- | --- | --- | --- | --- | --- | --- |
| TK1 | Women | 31 years old | Diploma | Midwife | Private Hospital | 10 years |
| TK2 | Men | 31 years old | Diploma | Nurse | Community Health Centers | 4 years |
| TK3 | Women | 30 years old | Diploma | Midwife | Hospital | 3 years |
| TK4 | Women | 23 years old | Doctor profession | Doctor | Community Health Centers | 1 year |
| TK5 | Women | 52 years old | Bachelor | Midwife | Community Health Centers | 30 years |

**Demographics of Respondents for Public at the Demonstration Stage in the Third Iteration**

| **Respondent** | **Gender** | **Age** | **Occupation** | **Age** |
| --- | --- | --- | --- | --- |
| MU1 | Women | 36 years old | Civil servant | 16 months |
| MU2 | Women | 30 years old | Private employee | 18 months |
| MU3 | Men | 24 years old | Private employee | - |
| MU4 | Women | 24 years old | Private employee | - |
| MU5 | Men | 22 years old | Private employee | - |
| MU6 | Men | 22 years old | Private employee | - |

**Demographics of Questionnaire Respondents at the Demonstration Stage in the Third Iteration**

| Demographic Variables | | Number | Percentage |
| --- | --- | --- | --- |
| Gender | Female | 158 | 51.8% |
|  | Male | 147 | 48.2% |
| Age | <19 years | 33 | 10.82% |
|  | 19-24 years | 154 | 50.49% |
|  | 25-34 years | 34 | 11.15% |
|  | 35-44 years | 18 | 5.9% |
|  | 45-54 years | 60 | 19.67% |
|  | >54 years | 6 | 1.97% |
| Education | Middle School or equivalent | 27 | 8.85% |
|  | High School or equivalent | 22 | 7.21% |
|  | Diploma | 13 | 4.26% |
|  | Bachelor | 197 | 64.59% |
|  | Masters | 42 | 13.77% |
|  | Doctor | 3 | 0.98% |
|  | Profession | 1 | 0.33% |
| Occupation | Student | 29 | 9.51% |
|  | College Student | 63 | 20.66% |
|  | Doctor | 2 | 0.66% |
|  | Nurse | 4 | 1.31% |
|  | IT | 39 | 12.79% |
|  | Entrepreneur | 12 | 3.93% |
|  | Housewife | 10 | 3.28% |
|  | Private employees | 58 | 19.02% |
|  | Civil servant | 64 | 20.98% |
|  | Doesn't work | 15 | 4.92% |
|  | Other | 9 | 2.95% |
| Domicile | Greater Jakarta | 140 | 45.90% |
|  | Java | 25 | 8.20% |
|  | Kalimantan | 4 | 1.31% |
|  | Sumatra | 134 | 43.93% |
|  | Sulawesi | 2 | 0.66% |

**General Improvement Evaluation Summary in the Third Iteration**

| Question | | Number | Percentage |
| --- | --- | --- | --- |
| Problems when trying the prototype | Yes | 47 | 15.41% |
|  | No | 258 | 84.59% |
| Ease of use level | Very easy | 62 | 20.33% |
|  | Easy | 156 | 51.15% |
|  | Normal | 83 | 27.21% |
| Prototype increase immunization knowledge | Yes | 287 | 94.1% |
|  | No | 18 | 5.9% |
| Applications can increase a person's desire to get immunized | Yes | 272 | 89.18% |
|  | No | 33 | 10.82% |
| Satisfied with the design of the prototype | Yes | 294 | 96.39% |
|  | No | 11 | 3.61% |
